# Impact of Climate Change on Potential Distribution of Chinese Caterpillar Fungus (*Ophiocordyceps sinensis*) in Nepal Himalaya

**DOI:** 10.1371/journal.pone.0106405

**Published:** 2014-09-02

**Authors:** Uttam Babu Shrestha, Kamaljit S. Bawa

**Affiliations:** 1 Institute for Agriculture and the Environment, University of Southern Queensland, Toowoomba, Queensland, Australia; 2 Department of Biology, University of Massachusetts, Boston, Massachusetts, United States of America; 3 Ashoka Trust for Research in Ecology and Environment (ATREE), Bangalore, India; Field Museum of Natural History, United States of America

## Abstract

Climate change has already impacted ecosystems and species and substantial impacts of climate change in the future are expected. Species distribution modeling is widely used to map the current potential distribution of species as well as to model the impact of future climate change on distribution of species. Mapping current distribution is useful for conservation planning and understanding the change in distribution impacted by climate change is important for mitigation of future biodiversity losses. However, the current distribution of Chinese caterpillar fungus, a flagship species of the Himalaya with very high economic value, is unknown. Nor do we know the potential changes in suitable habitat of Chinese caterpillar fungus caused by future climate change. We used MaxEnt modeling to predict current distribution and changes in the future distributions of Chinese caterpillar fungus in three future climate change trajectories based on representative concentration pathways (RCPs: RCP 2.6, RCP 4.5, and RCP 6.0) in three different time periods (2030, 2050, and 2070) using species occurrence points, bioclimatic variables, and altitude. About 6.02% (8,989 km^2^) area of the Nepal Himalaya is suitable for Chinese caterpillar fungus habitat. Our model showed that across all future climate change trajectories over three different time periods, the area of predicted suitable habitat of Chinese caterpillar fungus would expand, with 0.11–4.87% expansion over current suitable habitat. Depending upon the representative concentration pathways, we observed both increase and decrease in average elevation of the suitable habitat range of the species.

## Introduction

The climate of our planet is changing at an unprecedented rate. Global average temperature has increased by 0.85°C from 1880 to 2012 and it is likely to increase further by a minimum of 0.3°C–1.7°C (RCP 2.6) to a maximum of 2.6°C–4.8°C (RCP 8.5) by the end of this century relative to 1986–2005 temperature [1]. Ecosystems and species have already responded to global climate change [2]. Changes in community structure, composition, and dynamics have been observed at ecosystem levels [3]–[4] whereas alteration in phenology, modification of physiology, and shifts in distribution have been documented at the level of species [5]. Shifts in plant species distribution may increase vulnerability to extinction [6]. Therefore, understanding the potential impacts of climate change on the distribution of species is important for mitigation of future biodiversity losses [7].

The Himalaya, a region of unique biodiversity, rich cultural and ethnic diversity, and varied topography has warmed up by about 1.5°C, three times more than the global average, during the last 25 years from 1982 to 2006 [8]. Climate change in the Himalaya has already impacted hydrology, agriculture, ecosystems, and species [9]. Similarly, changes in phenology of vegetation [8] and altitudinal shifts in vegetation communities [10] and species such as *Rheum nobile*, *Saussurea stella*, *Rhodiola bupleuroides*, *Ponerorchis chusua*, *Microgynaecium tibeticum*, *Meconopsis simplicifolia*, and *Pedicularis trichoglossa* [11] have been noted. Comparatively higher resolution (50×50 km) regional climate models show that temperature and precipitation in the Himalayan region will continue to increase in future [12], and these changes are further likely to impact the distribution of biodiversity, as for example, predicted for Rhododendrons [13]. However, potential shift of many other plant species caused by past and future climate changes in this biologically diverse region of the world are largely unknown [11], [13].

Historical records have been largely used in documenting potential shifts in the distribution of species due to the past climate. However, in many cases, historical data on species distribution are lacking in the Himalaya. Moreover, such approaches might not be sufficient to predict the changes in current predicted distribution due to future changes in climate. Species distribution modeling (SDM), based on the quantitative relationship between environmental variables and species occurrence points, has made it possible to recognize species' niche requirements [14] and to understand the impacts of climate change on species distribution [15]. Various SDM methods, statistical, machine learning, and classification and distance are in current use to model species distribution [16]. Among them, a general purpose machine learning method—MaxEnt, developed by Phillips et al. (2006) [17]—is one of the most popular methods [18] and used to model a wide range of plant and animal species such as Malayan Sun Bear [19], Snow leopard [20] Lantana [21], Yew [22], Rhododendrons [13] and habitats globally [18]–[23]. It performs very well to estimate current as well as future distributions of species due to climate change [24].

Here we use the MaxEnt model to map the current potential distribution of Chinese caterpillar fungus (*Ophiocordyceps sinensis*) and to predict changes in the potential distribution of the species under future climate change trajectories in Nepal Himalaya. We also use the model to predict changes in the current potential elevation range of Chinese caterpillar fungus due to future climate change. We assume that both the extent of suitable habitat and range of Chinese caterpillar fungus will change with the change in climate in the area.

## Materials and Methods

### Study Area

Nepal, a mountainous country is situated on the southern slope of the central Himalaya. The country covers an area of 147,181 km^2^ and has the widest elevational gradient of any country in the world, ranging from 60 m.a.s.l (meters above sea level) to Mount Everest, the highest point of the world at 8,848 m.a.s.l.. This vast altitudinal gradient creates a wide variation in physiographic, climatic, topographic, and edaphic conditions resulting in rich biodiversity. The Chinese caterpillar fungus, which is reported from 27 mountain districts of Nepal, is one of the most important biological and socio-economic components of Nepal's rich biodiversity.

Nepal Himalaya has been experiencing climate change and its impact; mean annual maximum temperature increased by 0.6°C per decade during 1977–2000 [25]. Similarly, climate model projections for Nepal show temperature increases of 0.5–2.0°C (mean 1.4°C) by the 2030s; 1.7–4.1°C (mean 2.8°C) by the 2060s; and 3.0–6.3°C (mean 4.7°C) by the 2090s [26]. Although there is no clear trend in precipitation, different models show an increase in monsoon precipitation and a decrease in winter precipitation in the future [26].

### Species

Chinese caterpillar fungus is an endemic species of the Himalayan countries: Bhutan, China, India and Nepal. It a parasitic complex formed by a parasitic relationship between the fungus, *Ophiocordyceps sinensis* and the caterpillar of ‘ghost’ moth species belonging to the genus *Thitarodes* [27]. In Nepal, this caterpillar-fungus complex occurs in the open grasslands at elevations between 3,500–5,000 m.a.s.l. [28]–[29]. Although it has various therapeutic usages, the major trade of Chinese caterpillar fungus, with the popular name “Himalayan Viagra”, is due to its presumed effects as an aphrodisiac and powerful tonic [27]. It is one of the most expensive natural medical resources of the world [30]–[31]. In September 2012, the highest price of Chinese caterpillar fungus in China—the major trade destination—was $140,000/kg (for the best quality product), two times more expensive than gold [32]. Although the fungus has an extraordinarily high price, it is collected extensively by the poorest mountain communities to support their livelihood. In recent years, the availability of the Chinese caterpillar fungus in its natural habitat has been dwindling and may threaten the livelihoods of the poorest mountain people [29]. The decline has been attributed, in part, to climate change [27], [29], [33].

### Species location data

**Figure 1:**
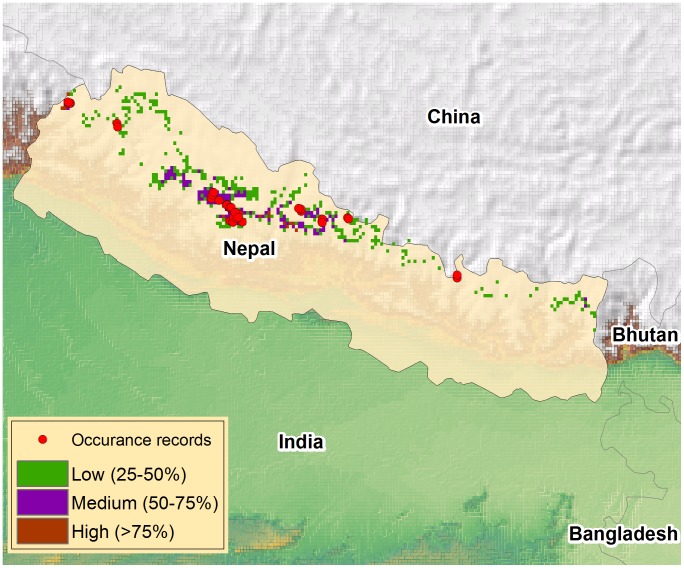
Predicted potential distribution of Chinese caterpillar fungus under current bioclimatic conditions and location of occurrence used for modeling.

The species occurrence data used for this study were collected from our own field surveys conducted in six districts (Dolpa, Manang, Dolakha, Gorkha, Darchula, Bajhang) of Nepal between 2011 and 2013. Altogether, 37 species occurrence points distributed in central, western and eastern Nepal were used to model the potential distribution (Figure 1). We secured the necessary permits from the National Trust for Nature Conservation (NTNC) to conduct field studies in protected areas of Manang and Gorkha.

### Environmental and bioclimatic data

To model potential distribution of Chinese caterpillar fungus across our study area, we first collected 19 bioclimatic layers, and one topographic (altitude) layer. The 19 grid-based bioclimatic variables (Table 1) were downloaded from the worldclim datasets (www.worldclim.com). Bioclimatic variables calculated from monthly temperature and precipitation values were generated through interpolation of average monthly climate data from weather stations at 2.5 arc minute spatial resolution [34]. They are ecologically meaningful variables that describe annual trends, seasonality, and extremes of temperature and precipitation. Altitude was derived from digital elevation data from the Shuttle Radar Topographic Mission (SRTM); this was then resampled into 2.5 arc minute (∼5 km) spatial resolution by using the nearest neighbor resampling technique in ArcGIS.

**Table 1:** Bioclimatic variables used for modeling habitat of Chinese caterpillar fungus.

Variables Name	Code	Data Source	Resolution
Altitude	Alt	STRM	90 m
Annual Mean Temperature	BIO 1	Worldclim	∼5 km (2.5 arc min)
Mean Diurnal Range [Mean of monthly (Max Temperature - Min Temperature)]	BIO 2	Worldclim	∼5 km (2.5 arc min)
Isothermality (BIO 2/BIO 7) (*100)	BIO 3	Worldclim	∼5 km (2.5 arc min)
Temperature Seasonality (Standard Deviation*100)	BIO 4	Worldclim	∼5 km (2.5 arc min)
Max Temperature of Warmest Month	BIO 5	Worldclim	∼5 km (2.5 arc min)
Min Temperature of Coldest Month	BIO 6	Worldclim	∼5 km (2.5 arc min)
Temperature Annual Range (BIO 5-BIO 6)	BIO 7	Worldclim	∼5 km (2.5 arc min)
Mean Temperature of Wettest Quarter	BIO 8	Worldclim	∼5 km (2.5 arc min)
Mean Temperature of Driest Quarter	BIO 9	Worldclim	∼5 km (2.5 arc min)
Mean Temperature of Warmest Quarter	BIO 10	Worldclim	∼5 km (2.5 arc min)
Mean Temperature of Coldest Quarter	BIO 11	Worldclim	∼5 km (2.5 arc min)
Annual Precipitation	BIO 12	Worldclim	∼5 km (2.5 arc min)
Precipitation of Wettest Month	BIO 13	Worldclim	∼5 km (2.5 arc min)
Precipitation of Driest Month	BIO 14	Worldclim	∼5 km (2.5 arc min)
Precipitation Seasonality (Coefficient of Variation)	BIO 15	Worldclim	∼5 km (2.5 arc min)
Precipitation of Wettest Quarter	BIO 16	Worldclim	∼5 km (2.5 arc min)
Precipitation of Driest Quarter	BIO 17	Worldclim	∼5 km (2.5 arc min)
Precipitation of Warmest Quarter	BIO 18	Worldclim	∼5 km (2.5 arc min)
Precipitation of Coldest Quarter	BIO 19	Worldclim	∼5 km (2.5 arc min)

We calculated pairwise correlations and removed highly correlated variables (r^2^≤0.80) to minimize the impact of multicollinearity and over-fitting of the model. The remaining seven (Bio 3, Bio 7, Bio 11, Bio 14, Bio 15, Bio 18, and Bio 19) bioclimatic variables (Table 2) were used to model the distribution of Chinese caterpillar fungus in current and future climate conditions. All datasets were changed into ASCII files in ArcGIS as required by the MaxEnt software. We repeated the same process to generate predicted maps in three different future climate scenarios for 2030, 2050 and 2070. We did not take account of land use or land cover change, human disturbances, species dispersal or changes in biotic interactions in the model.

**Table 2:** Correlation matrix of altitude and bioclimatic variables.

Code	Alt	BIO1	BIO2	BIO3	BIO4	BIO5	BIO6	BIO7	BIO8	BIO9	BIO10	BIO11	BIO12	BIO13	BIO14	BIO15	BIO16	BIO17	BIO18	BIO19	LULC
Alt		−1.00	−0.06	0.13	0.10	−0.99	−0.99	−0.14	−1.00	−0.99	−1.00	−1.00	−0.76	−0.81	0.00	−0.74	−0.79	0.31	−0.58	0.46	0.12
BIO1			0.05	−0.14	−0.10	0.99	0.99	0.13	1.00	0.99	1.00	1.00	0.76	0.80	0.01	0.73	0.78	−0.28	0.57	−0.44	−0.13
BIO2				0.08	0.73	0.15	−0.08	0.88	0.08	0.00	0.09	0.01	−0.20	−0.07	−0.51	0.40	−0.10	−0.44	−0.27	−0.35	0.18
**BIO3**					**−0.54**	**−0.23**	**−0.13**	**−0.40**	**−0.15**	**−0.15**	**−0.17**	**−0.11**	**0.05**	**0.01**	**−0.07**	**−0.01**	**0.04**	**−0.49**	**0.30**	**−0.49**	**0.01**
BIO 4						0.03	−0.21	0.93	−0.07	−0.14	−0.05	−0.16	−0.35	−0.24	−0.32	0.15	−0.27	0.04	−0.52	0.14	0.17
BIO5							0.97	0.26	0.99	0.98	0.99	0.98	0.70	0.76	−0.02	0.73	0.74	−0.24	0.48	−0.38	−0.11
BIO6								0.01	0.98	0.99	0.98	0.99	0.78	0.80	0.09	0.65	0.79	−0.20	0.60	−0.37	−0.16
**BIO7**									**0.16**	**0.09**	**0.18**	**0.08**	**−0.19**	**−0.05**	**−0.43**	**0.38**	**−0.09**	**−0.16**	**−0.37**	**−0.09**	**0.15**
BIO8										0.99	1.00	1.00	0.75	0.80	0.00	0.74	0.78	−0.29	0.57	−0.45	−0.12
BIO9											0.99	0.99	0.76	0.80	0.02	0.69	0.78	−0.26	0.58	−0.41	−0.13
BIO10												0.99	0.74	0.79	−0.01	0.74	0.77	−0.28	0.55	−0.43	−0.12
**BIO11**													**0.77**	**0.80**	**0.03**	**0.71**	**0.79**	**−0.28**	**0.60**	**−0.44**	**−0.13**
BIO12														0.98	0.18	0.63	0.99	−0.22	0.92	−0.38	−0.16
BIO13															0.06	0.74	0.99	−0.32	0.88	−0.47	−0.13
**BIO14**																**−0.33**	**0.07**	**0.62**	**0.14**	**0.46**	**−0.14**
**BIO15**																	**0.72**	**−0.64**	**0.53**	**−0.73**	**−0.06**
BIO16																		−0.31	0.90	−0.46	−0.14
BIO17																			−0.32	0.96	−0.13
**BIO18**																				**−0.46**	**−0.14**
**BIO19**																					**−0.08**

Unrelated variables (Correlation ≤0.80) used for the study BIO3, BIO7, BIO 11, BIO 15, BIO 18, BIO 19, LULC.

To determine the future distribution of the species under different climate trajectories, we used datasets of future climate from the International Center for Tropical Agriculture (www.ccafs-climate.org), a global agricultural research institution. We selected three future GHG (greenhouse gas) concentration trajectories, also known as representative carbon pathways (RCP 2.6, RCP 4.5 and RCP 6.0), for three different time periods (2030, 2050 and 2070) as adopted by the IPCC in its fifth Assessment Report (AR5) [1]. RCP 2.6 is the lowest GHG concentration pathway in which radioactive forcing (global energy imbalances) levels reach 3.1 W/m^2^ by mid-century and drops 2.6 W/m^2^ by 2100 [35]. RCP 4.5 is a stabilization scenario in which the total radiative forcing reaches to 4.5 W/m^2^ by 2100 and stabilizes due to the employment of a range of technologies and strategies for reducing GHG emissions [36]. Likewise, RCP 6.0 also represents stabilization by 2100, this time at 6.0 W/m^2^ by 2100 [37]. We selected a global circulation model, HadGEM2-CC (Hadley Global Environment Model 2 Carbon Cycle) developed by the Hadley Center, United Kingdom [38]. HadGEM2 models have been used to perform all the CMIP5 (Coupled Model Intercomparison Project Phase 5) centennial experiments including ensembles of simulations of the RCPs. HadGEM2-CC is one of the models used by the international governmental panel on climate change (IPCC) in its fifth Assessment Report (AR5). For consistency, we used the same seven bioclimatic variables (Bio 3, Bio 7, Bio 11, Bio 14, Bio 15, Bio 18, and Bio 19) we used for modeling current potential distribution of the species to predict future distribution. Those data are statistically downscaled from a Global Circulation Model (GCM) based on the sum of interpolated anomalies to high resolution monthly climate surfaces from Worldclim [34].

### Modeling

We used freely available MaxEnt software to model the current and future distributions of Chinese caterpillar fungus. MaxEnt is a general purpose machine learning method that estimates the probability distribution of a species occurrence based on environmental conditions of a location in which the species is found by calculating the distribution of maximum entropy i.e. the most spread out distribution in space for a given set of constraints [17]. It is the most popular species distribution modeling method with more than 1000 published usages since 2005 [18], [23]. MaxEnt has also outperformed other methods and has shown higher predictive accuracy than other methods [6], [39]. MaxEnt performs well to estimate potential range shifts of species due to climate change [24]. The method is easy to use and has the functionality to use presence only data.

We used the following parameter values in the MaxEnt model: random test percentage  = 25%, regularization multiplier  = 1, maximum number of backgrounds points  = 10,000, maximum iterations  = 1,000 and convergence threshold  = 0.00001. This means that the model uses 25% of the original presence data against 10,000 random background points (pseudo-absences) for modeling the distribution. Model robustness is commonly evaluated by AUC values that range from 0 to 1; AUC values between 0.5–0.7 are considered low, 0.7–0.9 moderate and >0.9 high [16], [35]. We also used AUC values to determine the model accuracy. The output of MaxEnt is continuous data with values ranging from 0 (lowest) to 1 (highest) probability of distribution. We imported the MaxEnt output data and reclassified into three classes of habitat suitability: low suitability (25–50% probability of occurrence), medium suitability (50–75% probability of occurrence) and high suitability (>75% probability of occurrence) by omitting the values below 25% as non-suitable habitat based on the logistic threshold, with suitable conditions predicted above the threshold and unsuitable below. This reclassification of suitable conditions allowed us to compare the change in classes over time and space.

We also estimated the total area of predicted habitat of Chinese caterpillar fungus under current and future climatic conditions by calculating the number of ‘presence’ grid cells multiplied by their spatial resolution. To observe potential changes in the altitudinal distribution of Chinese caterpillar fungus, we extracted elevation values of the pixels of predicted presence from the predicted and current distribution maps generated by MaxEnt models. We compared the mean elevation values of the areas of predicted presence for current and future climate scenarios using independent sample T-test.

## Results

**Figure 2:**
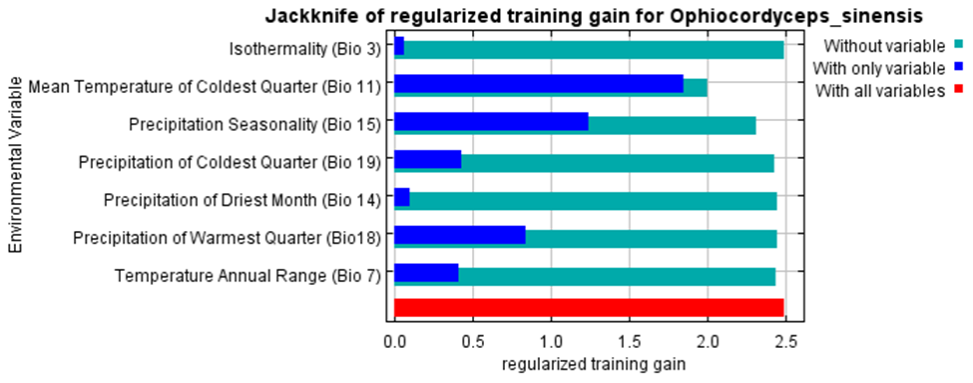
Results of jackknife test of relative importance of predictor variables for Chinese caterpillar fungus.

Our study demonstrates for the first time the potential distribution of Chinese caterpillar fungus habitat in Nepal Himalaya. Out of seven predictor bioclimatic variables used for this study, the relative contribution of two variables (Bio 11 and Bio 15) to the model was more than 89%. Mean temperature of the coldest quarter (Bio 11) had the highest contribution (74.5%) to the model followed by precipitation seasonality (Bio 15), with 15.3% contribution. The bioclimatic variable, isothermality (Bio 3) had the lowest contribution (0.3%). The MaxEnt model's jack-knife test of variable importance showed that the mean temperature of the coldest quarter (Bio 11) was the variable with the highest gain when used in isolation and the gain decreased when it was omitted (Figure 2). The validity of the model for current distribution of Chinese caterpillar fungus was high with AUC = 0.98 indicating that the selected variables described the distribution of Chinese caterpillar fungus very well.

**Figure 3:**
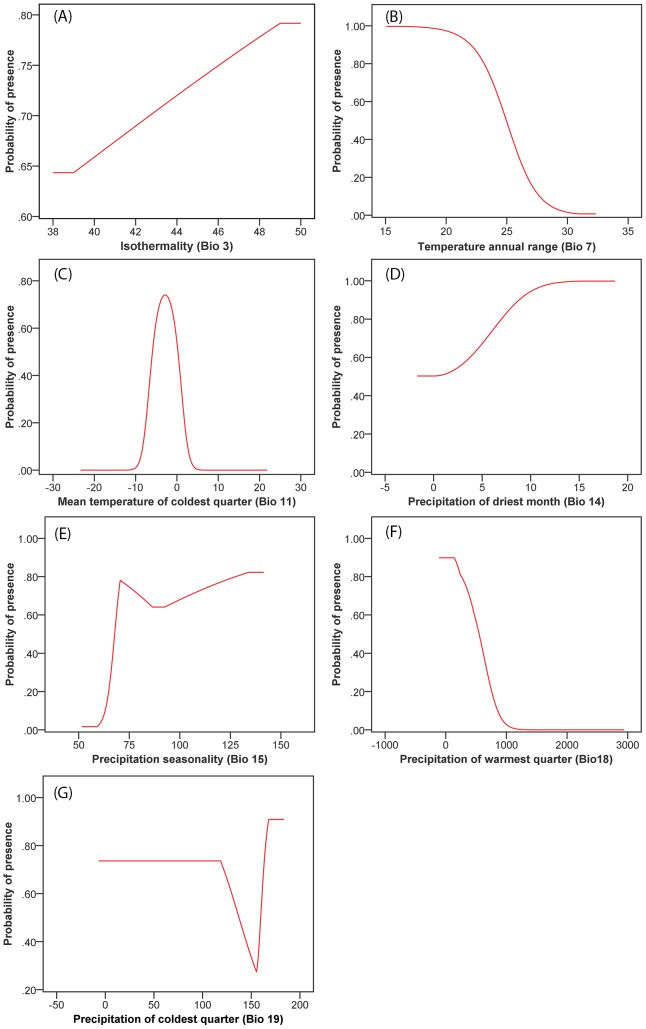
Response curves for the predictors of the MaxEnt Model.

Responses of each environmental variable that influenced the predicted suitable distribution of Chinese caterpillar fungus are shown in response curves (Figure 3). These response curves show changes in the logistic prediction when each environmental variable changes by keeping all other environmental variables at their average sample value. Models showed that the distribution of Chinese caterpillar fungus is highly controlled by temperature. It prefers areas where the mean annual temperature remains below 15°C and the mean temperature of the coldest quarters stays slightly lower than zero but not less than minus 10°C. Precipitation in the driest month and precipitation seasonality from 75–150 mm might also be useful predictors but excessive precipitation above 200 mm was not found to be very apposite.

The potential distribution of Chinese caterpillar fungus under current bioclimatic conditions and occurrence locations is shown in Figure 1. Based on our model, about 6.02% (8,989 km^2^) of the country is suitable for Chinese caterpillar fungus habitat, and the fungus occurs in 26 mountainous districts of Nepal. The greatest concentration of pixels (potential habitat or predicted presence) is observed in Dolpa, Rukum, Manang, Myagdi, and Jumla districts when the suitable habitat map is overlaid with the district map of Nepal.

**Figure 4:**
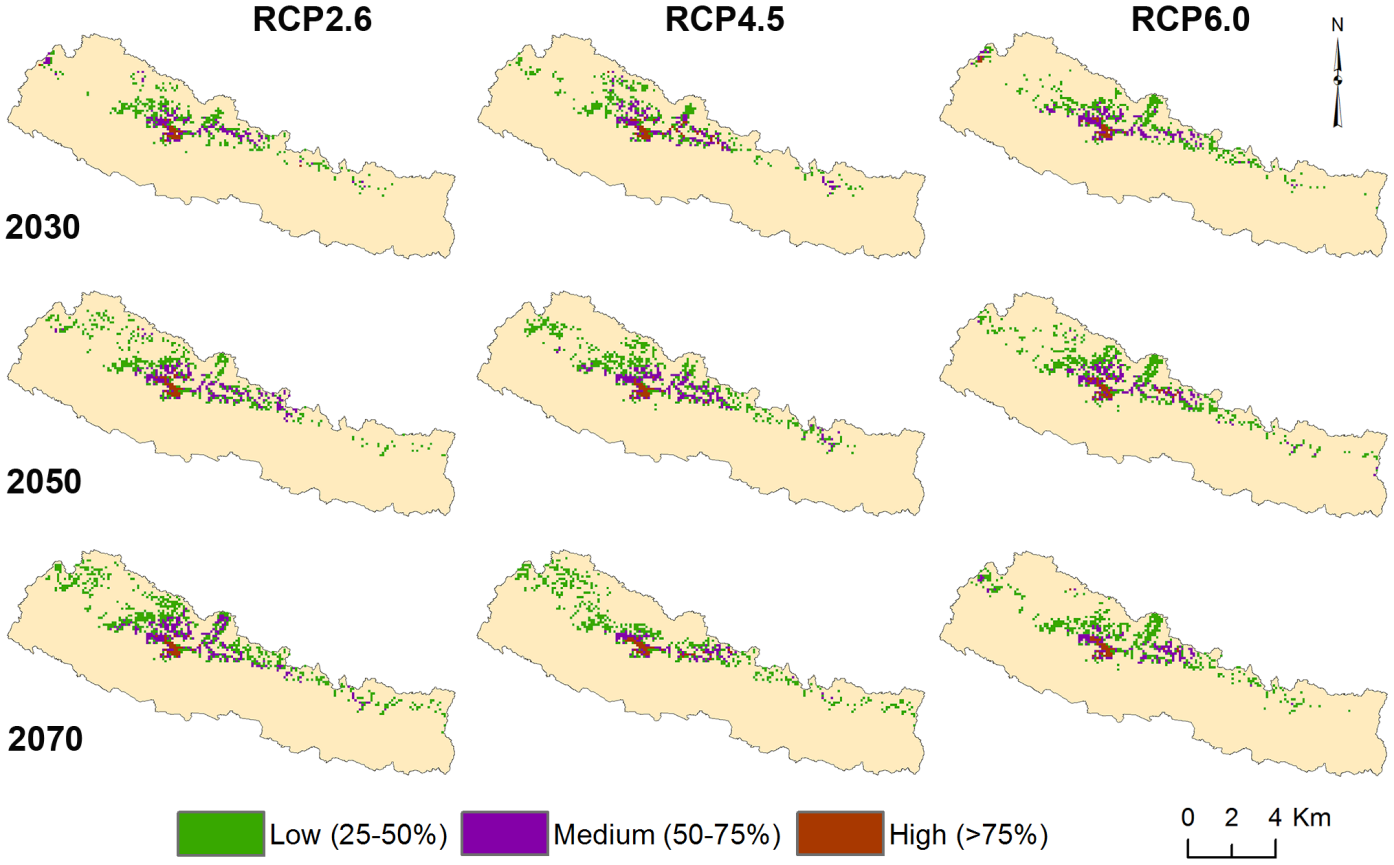
Predicted future distribution of Chinese caterpillar fungus in future climate scenarios.

**Figure 5:**
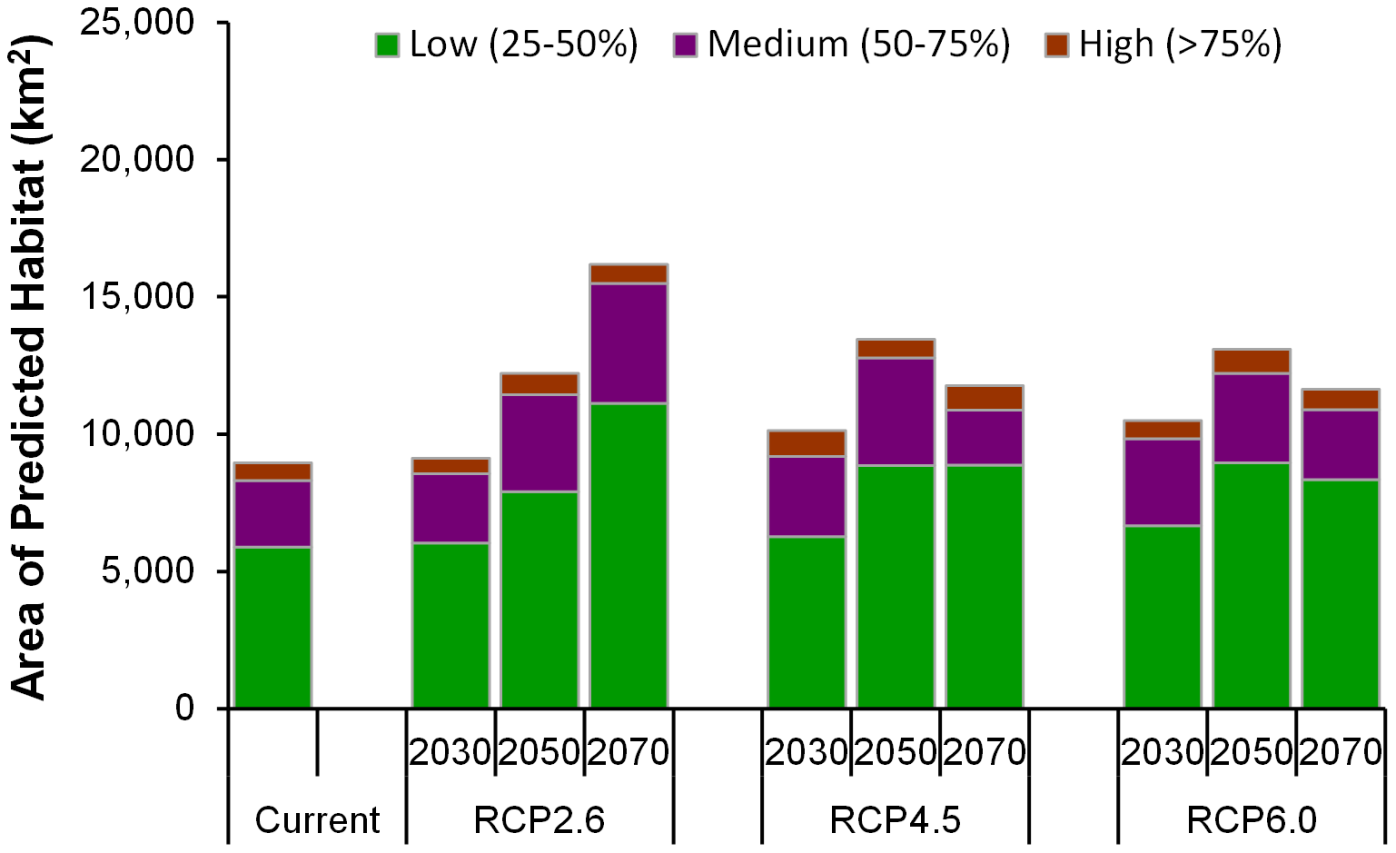
The estimated areas of the predicted distribution of Chinese caterpillar fungus.

To estimate the potential impact of climate change on the distribution of Chinese caterpillar fungus, the predicted distribution of Chinese caterpillar fungus in future climate scenarios is shown in Figure 4. Across all scenarios in three different periods, the predicted area of Chinese caterpillar fungus distribution was predicted to increase. The estimated area of the predicted distribution is given in Figure 5. The maximum expansion (a 4.87% addition to the current potential suitable area) would occur under RCP 2.6 by 2070 whereas the minimum expansion (0.11% addition in current potential suitable area) was predicted for the year 2030 for the same pathway. Much of the expansion occurs in western Nepal whereas there are nominal changes only in the eastern parts of the country. To be precise, maximum increases in the area of suitable habitat occurs in Dolpa, Mustang, and Mugu districts under RCP 2.5 in 2050 whereas the greatest reduction in habitat area occurs in the Taplejung district of Eastern Nepal. Taplejung might lose its entire area of suitable habitat under RCP 4.5 by the year 2050. Similar loss of the entire area of Chinese caterpillar fungus habitat was predicted in Humla district under RCP 6.0 by the year 2070. However, expansion of potential habitat might occur in two new districts, Dolpa and Pachthar under RCP 6.0 by 2070. Under both the RCP 4.5 and RCP 6.0 trajectories, the area of predicted distribution increases continuously up to 2050 then decreases by 2070 whereas in the RCP 2.6 trajectory, the distribution continuously increases from 2030 to 2070 and reaching a maximum area in 2070 (Figure 5).

**Figure 6:**
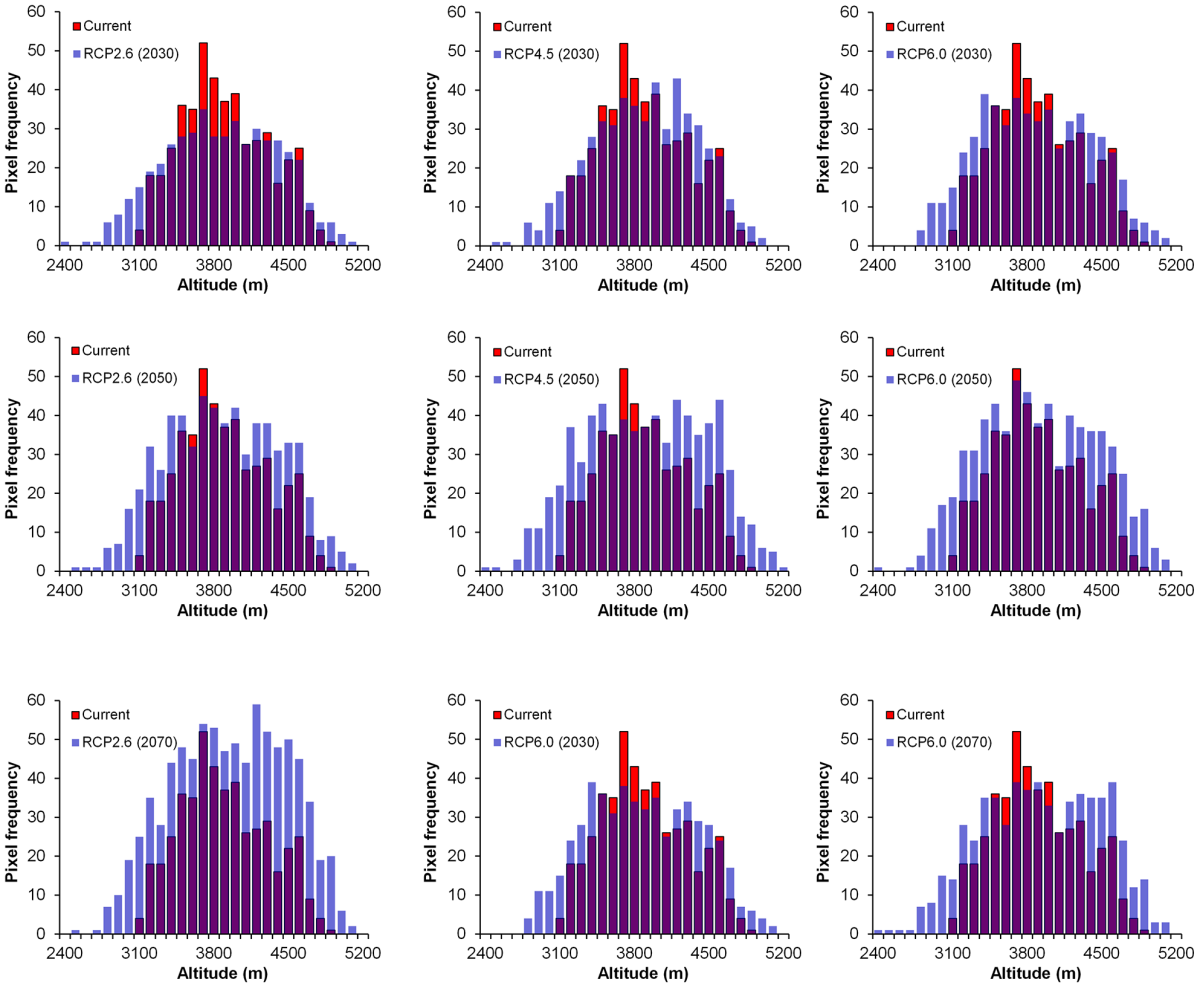
Change in average elevation in distribution of Chinese caterpillar fungus in three future climate scenarios.

Expansion in both lower and upper altitudinal limits was observed under all future climate change pathways (Figure 6). The average altitude of current distribution and that of future distributions increased under four pathways: RCP 2.6 (2070), RCP 4.5 (2050), RCP 6.0 (2050) and RCP 6.0 (2070) whereas a decrease was found in all other pathways (Table 3). However, none of the changes were statistically significant (p = 0.01) except RCP 4.5 (2070) based on the independent sample T-test between the altitude means of current potential distribution range and that of future potential distribution range.

**Table 3:** Independent sample T-test between average altitude of current predicted distribution and future predicted distributions.

Climate trajectories	Year	Mean	SD	P value of independent sample T-test
RCP2.6	2030	3921.68	523.03	0.302
RCP2.6	2050	3933.59	523.11	0.500
RCP2.6	2070	3998.86	529.83	0.104
RCP4.5	2030	3948.13	486.09	0.861
RCP4.5	2050	3955.49	566.19	0.938
RCP4.5	2070	3878.31	525.48	0.011
RCP6.0	2030	3929.22	514.55	0.416
RCP6.0	2050	3959.01	538.16	0.841
RCP6.0	2070	3982.11	548.55	0.337

## Discussion

Our work represents the first attempt to model the impact of future climate change on the distribution of Chinese caterpillar fungus—a flagship species of the Himalaya. Climate change projections for the Nepal Himalaya predict that average annual temperatures will increase by 3.0–6.3°C (mean 4.7°C) by the 2090s and that the warming would be greater in higher elevation regions—habitats of the Chinese caterpillar fungus [26]. Since climate change has already impacted species' habitats including those of several species of fungi worldwide [40]–[42], it will likely affect the distribution of this species in Nepal as well. We here provide a model that could be used to predict how the distribution of species might be affected by changes in future climate depending upon the different representative concentration pathways.

It is widely believed that temperature and humidity play important roles in yields [27]–[28], abundance [43], and the probability of infection and sporulation [44] of Chinese caterpillar fungus. Furthermore, Chinese caterpillar fungus distribution is also assumed to be affected by winter and summer temperatures and the seasonality of precipitation [45]–[46]. Our observations that the mean temperature of the coldest quarter and the seasonality of precipitation affect the distribution of Chinese caterpillar fungus is in line with observations that temperature and humidity play important roles in the ecology and physiology of the fungus. Furthermore, bioclimatic response variables that affect the predicted distribution of the species concur with the knowledge of local communities about the habitat needs of the species [46].

Although the potential distribution predicted by the model is not the observed distribution, there is close congruence between the two distributions. The districts of Dolpa, Rukum, Manang, Myagdi, and Jumla showed a greater area of potential distribution (maximum concentration of the pixels). These districts are the major producers of Chinese caterpillar fungus in Nepal, contributing approximately 60% of the total volume of Chinese caterpillar fungus traded in Nepal [47]. Spatial overlap of the potential occurrence with districts where Chinese caterpillar fungus is reported is 96.29% (26 districts in the model vs. 27 districts in actual). However, the model slightly overestimates the potential distribution for the Kaski district and underestimates it for Dolakha District. Nonetheless, both the maximum spatial overlapping and high AUC value suggests that the model has a high level of accuracy.

Our models show that the potential distribution of Chinese caterpillar fungus will extend under all future climate scenarios, suggesting that additional new suitable habitats will be created for Chinese caterpillar fungus with the future climate change. However, this expansion may not guarantee an increase in the production of Chinese caterpillar fungus in future. Studies in other parts of the world have shown both expansion and shrinkage of potential habitat in response to future climate change [48]–[50]. Both the reductions and increase in range size were predicted for the endemic flora of California under future climate change scenarios [48] as well as higher plants in Europe [49]. Range expansion of lemur parasites is predicted for Madagascar [50] whereas shrinkage of Rhododendrons has been predicted in the Himalaya [13]. Likewise a 60% reduction in the range of Ngwayir (*Pseudocheirus occidentalis*) is predicted in Australia [51].

It has been unofficially reported that the altitudinal limit of Chinese caterpillar fungus seems to have shifted upwards by 200–500 m in China due to past climate change [27], [33], which may have also caused local extinction of the fungus from Mount Emai in the Chinese province of Sichuan, located on the edge of the Tibetan plateau [52]–[53]. However, we found no noteworthy changes in the altitudinal ranges of the modeled potential distributions of Chinese caterpillar fungus under predicted future climate change trajectories in this study. Furthermore, it is unclear what methodology Yang (2008) had used to observe the shifts in the altitudinal range of Chinese caterpillar fungus [33]. Due to the unavailability of historical data on the distribution of Chinese caterpillar fungus in Nepal Himalaya, monitoring the impact of past climate change on the distribution of Chinese caterpillar fungus is impossible.

One of the major current drivers of decline of Chinese caterpillar fungus is extensive harvesting [27], [29]. Every year about 100 thousand people are involved in harvesting Chinese caterpillar fungus in Nepal [54]. The abundance of Chinese caterpillar fungus in the future will largely depend on human use. Although the area of suitable habitat for Chinese caterpillar fungus is predicted to expand with future climate change, the availability of fungus is likely to be largely governed by human impacts on populations. Future distribution models will have to take into account actual and potential harvesting intensities at different locations. Our model does not account for harvesting pressure or future transformation of land. Furthermore, since the Chinese caterpillar fungus complex involves an array of linked biotic interactions of diverse organisms with different life cycles and generation spans, future climate change may affect the interacting species differentially. Future research would therefore need to incorporate harvesting, future land use and land cover change, and biotic interactions in distribution models of not only this species but other species as well.

Although this study lays down the foundation for studying the dynamics of Chinese caterpillar fungus distribution through time and space, it has some limitations. Bioclimatic variables used in this study have a 5×5 km spatial resolution. This resolution may be acceptable for species with broad range sizes including Chinese caterpillar fungus [55], however, given the diverse topography of the Himalayan region, finer scale resolution of the environmental data might be needed to produce results at scales relevant to species with more restricted habitat requirements. As we used a limited number of samples from only six districts while this species is reported from 27 mountain districts of Nepal, this resolution was used as a compromise between fine scale resolution and the precision of the coordinates for the species' occurrences. More occurrence records spread across the entire country of Nepal and finer resolution of environmental data that also capture microclimates, edaphic conditions, vegetation dynamics, and landscape heterogeneity might enable more sophisticated models to be developed in future.

Despite these limitations, the study provides information on the suitable climate space or bioclimatic envelope responsible for the potential distribution of Chinese caterpillar fungus. Furthermore, understanding the potential distribution of one of the most exploited species of this region is a necessary to design an efficient conservation plan. This study provides evidence of the distribution of suitable habitat for the Chinese caterpillar fungus in Nepal Himalaya using a robust bioclimatic model. It can be further useful in assessing threats and evaluating gaps in protected areas in order to conserve this economically important species. Furthermore, our evaluation of the potential impacts of future climate change on the potential suitable habitat of the species is important in devising adaptive responses and precautionary measures for the sustainable management of the habitat of the species in future.
